# Prognostic Value of Pan-Immune Inflammation Value for Major Adverse Cardiac Events and Mortality in Patients with Aortic Stenosis After TAVI

**DOI:** 10.3390/medicina61060992

**Published:** 2025-05-27

**Authors:** Mehmet Nail Bilen, Mehmet Rasih Sonsöz, Yusuf İnci, Yeliz Güler, Ahmet Güler, Hamdi Püşüroğlu

**Affiliations:** Department of Cardiology, Basaksehir Cam and Sakura City Hospital, University of Health Sciences, Istanbul 34000, Turkey; mrsonsoz@gmail.com (M.R.S.); dryusufinci@gmail.com (Y.İ.); yelizguler829@gmail.com (Y.G.); ahmetguler01@yahoo.com.tr (A.G.); hpusts@gmail.com (H.P.)

**Keywords:** aortic stenosis, pan-immune inflammation value, transcatheter aortic valve implantation, major cardiac events, mortality

## Abstract

*Aims:* This study aimed to evaluate whether the pan-immune inflammation value (PIV) has prognostic value for major adverse cardiac events (MACEs), including stroke, rehospitalization, and in-hospital and one-year all-cause mortality, in patients with aortic stenosis (AS) undergoing transcatheter aortic valve implantation (TAVI). *Methods:* A total of 152 patients undergoing TAVI were retrospectively analyzed and stratified into two groups based on a PIV cutoff value of 488. Baseline clinical, laboratory, echocardiographic, and procedural characteristics were compared. Clinical outcomes, including mortality, cerebrovascular events, and bleeding complications, were assessed. Multivariable logistic regression and receiver operating characteristic (ROC) curve analyses were performed to identify independent mortality predictors and evaluate the predictive performance of PIV. *Results:* Among the 152 patients (mean age 77 ± 7 years; 59.9% female), 52 (34.2%) had a PIV ≥ 488. These patients had significantly higher rates of diabetes mellitus (62% vs. 38%, *p* = 0.006), chronic kidney disease (31% vs. 12%, *p* = 0.005), and chronic obstructive lung disease (31% vs. 15%, *p* = 0.022), along with higher STS scores (16.3 vs. 11.7, *p* = 0.003). Inflammatory markers were elevated, and lymphocyte and hemoglobin levels were reduced in the high PIV group (*p* < 0.001). Patients with PIV ≥ 488 experienced significantly higher one-year mortality (58% vs. 4%, *p* < 0.001), in-hospital mortality (21% vs. 2%, *p* < 0.001), rehospitalization (29% vs. 4%, *p* < 0.001), ischemic cerebrovascular events (12% vs. 4%, *p* < 0.001), and major bleeding (10% vs. 2%, *p* = 0.034). Multivariable analysis identified age (OR: 1.108; 95% CI: 1.010–1.217; *p* = 0.031) and PIV (OR: 1.006; 95% CI: 1.003–1.008; *p* < 0.001) as independent mortality predictors. The PIV showed a strong predictive performance (AUC: 0.90, *p* < 0.001), with 88% sensitivity and 81% specificity. Kaplan–Meier analysis showed significantly lower survival in the high PIV group (*p* < 0.001). *Conclusions:* A high preprocedural PIV is an independent predictor of MACEs, in-hospital, and one-year mortality in AS patients undergoing TAVI.

## 1. Introduction

Aortic stenosis (AS) is a common and life-threatening valvular heart disease, particularly affecting elderly individuals. It is characterized by the progressive calcification of the aortic valve and the obstruction of the left ventricular outflow tract, which, if left untreated, can lead to heart failure, arrhythmias, or sudden cardiac death [1]. Over the past two decades, transcatheter aortic valve implantation (TAVI) has revolutionized the management of symptomatic severe AS, especially in patients considered at high or prohibitive surgical risk [2]. Despite the proven benefits of TAVI in reducing mortality and improving functional outcomes, a substantial proportion of patients continue to experience post-procedural complications, including major adverse cardiac events (MACEs) such as myocardial infarction, stroke, heart failure exacerbation, and elevated long-term mortality rates [3]. As such, identifying reliable biomarkers for risk stratification remains critical for optimizing post-TAVI management.

Growing evidence suggests that inflammation plays a key role in the pathogenesis of atherosclerosis, valvular calcification, and post-TAVI outcomes [4,5]. Among inflammatory markers, the neutrophil-to-lymphocyte ratio (NLR) has been widely recognized as a prognostic indicator in cardiovascular diseases [6,7]. More recently, a novel biomarker called the pan-immune inflammation value (PIV) has been introduced. The PIV combines neutrophil, platelet, monocyte, and lymphocyte counts to provide a comprehensive assessment of systemic immune response and inflammatory burden [PIV = Neutrophils × Monocytes × Platelets/Lymphocytes]. While elevated PIV levels have been explored in oncology and cardiovascular research, their prognostic value in the context of TAVI has not yet been investigated [8,9].

The present study aims to evaluate the prognostic significance of pre-procedural PIV in predicting MACEs and all-cause mortality in patients with severe AS undergoing TAVI. By examining the relationship between baseline PIV levels and post-TAVI clinical outcomes, this study seeks to determine whether PIV can serve as an effective tool for early risk stratification and clinical decision making. A better understanding of the role of systemic inflammation in post-TAVI prognosis may contribute to the development of personalized therapeutic strategies, ultimately improving long-term outcomes in this high-risk patient population.

## 2. Materials and Methods

### 2.1. Study Population

After ethics committee approval, 152 consecutive patients diagnosed with symptomatic severe AS who underwent TAVI at our institution between May 2021 and May 2023 were included in this retrospective, observational study. The diagnosis of severe AS was based on echocardiographic criteria, defined as an aortic valve area (AVA) ≤ 1.0 cm^2^ and/or a mean transaortic gradient ≥ 40 mmHg, in accordance with current American College of Cardiology/American Heart Association guidelines. Patients were deemed suitable candidates for TAVI based on a multidisciplinary Heart Team assessment [1].

Exclusion criteria included: (1) active infection or inflammatory disease at the time of the procedure, (2) hematologic disorders affecting leukocyte, platelet, or hemoglobin levels, (3) chronic immunosuppressive therapy or active malignancy, (4) missing or incomplete laboratory data necessary for calculating the PIV. Patients with severe AS were considered as a candidate for TAVI after being determined as a high or very high cardiac surgical risk. Procedural complications were defined using valve academic research consortium 3 (VARC-3) criteria [10].

#### Data Collection and Clinical Endpoints

Baseline demographic characteristics, medical history, and comorbid conditions such as chronic kidney disease, chronic obstructive pulmonary disease, and prior cardiovascular events were documented. Pre-procedural risk stratification was assessed using the Society of Thoracic Surgeons (STS) score. Procedural details, including valve type (self-expanding vs. balloon-expandable), vascular access route, and the need for post-TAVI pacemaker implantation, were recorded.

MACEs were defined as stroke or transient ischemic attack (TIA), rehospitalization, and short-term all-cause mortality within a 12-month period. Hospital electronic databases and statewide death registry databases were employed to assess and verify medium-term mortality. A stroke was classified as a TIA if the neurologic deficit lasted less than 24 h, and as a stroke if the deficit persisted for a longer duration. Rehospitalization was defined as any rehospitalization occurring within 30 days.

### 2.2. Transthoracic Echocardiography

M-mode and 2D ECHO were performed in the left lateral decubitus position using a 3.25 probe from the Vivid 5 ECHO echocardiography device, according to the American Society of Echocardiography criteria [11]. Parasternal short–long axis images and apical 4 and 2 chamber views, which are standard echocardiography positions, were used for measurements. Left ventricular ejection fraction was calculated using the modified Simpson’s method [12].

#### Laboratory Assessments and Calculation of the Pan-Immune Inflammation Value

Blood samples were collected from all patients within 24 h prior to the TAVI procedure. Laboratory analyses included measurements of total leukocyte count, neutrophils, lymphocytes, monocytes, platelets, hemoglobin levels, and C-reactive protein (CRP). Subsequently, the PIV was calculated.

### 2.3. Statistical Analysis

Statistical analyses were performed using SPSS Statistics (version 29, IBM, New York, NY, USA). Continuous variables were assessed for normality using the Kolmogorov–Smirnov test and are presented as mean ± standard deviation or median [interquartile range], as appropriate. Categorical variables are expressed as frequencies and percentages. Comparisons between groups were conducted using the independent sample’s *t*-test or Mann–Whitney U test for continuous variables, and the chi-square test for categorical variables.

Receiver operating characteristic (ROC) curve analysis was performed to evaluate the prognostic significance of the PIV in predicting all-cause mortality. Based on the ROC curve, an optimal PIV cutoff value of 488 was determined to maximize sensitivity and specificity. Patients were subsequently categorized into two groups according to this cutoff (PIV ≥ 488 vs. PIV < 488).

To identify independent predictors of all-cause mortality, variables with a *p* value < 0.1 in univariable analysis were included in a multivariable logistic regression model. Results are reported as odds ratios (OR) with 95% confidence intervals. The Kaplan–Meier method was used to estimate survival probabilities over time. Patients were stratified into groups based on PIV, and survival curves were generated for each group. The log-rank test was applied to compare survival distributions between groups. A *p* value < 0.05 was considered statistically significant.

## 3. Results

A total of 152 patients undergoing TAVI were included in this study, with a mean age of 77 ± 7 years and 59.9% being female. The patients were stratified according to the PIV cutoff of 488. Among the cohort, 52 patients (34.2%) had a PIV ≥ 488, and 100 patients (65.8%) had a PIV < 488.

Baseline demographic and clinical characteristics are presented in Table 1. There was no significant difference in age, sex distribution, body mass index, hypertension, or history of coronary artery disease between the two groups. However, patients with a PIV ≥ 488 had a significantly higher prevalence of diabetes mellitus (62% vs. 38%, *p* = 0.006), chronic kidney disease (31% vs. 12%, *p* = 0.005), and chronic obstructive lung disease (31% vs. 15%, *p* = 0.022). These patients also exhibited higher surgical risk scores, reflected by elevated STS scores (16.3 [10.8–19.7] vs. 11.7 [9.5–15.2], *p* = 0.003). Echocardiographic parameters, including left ventricular ejection fraction, transaortic mean gradient, and aortic valve area, were similar between the groups.

The laboratory parameters are summarized in Table 2. The patients with a PIV ≥ 488 demonstrated significantly higher levels of leucocytes, neutrophils, monocytes, platelets, glucose, urea, creatinine, and C-reactive protein, along with lower lymphocyte counts and hemoglobin levels (all *p* < 0.001, except urea *p* = 0.050 and creatinine *p* = 0.002). The median PIV was markedly elevated in patients with a PIV ≥ 488 group (760 (578–1041) vs. 246 (165–339), *p* < 0.001).

Medications, procedural features, and adverse events are presented in Table 3. There was no significant difference in age, sex distribution, body mass index, hypertension, or history of coronary artery disease between the two groups. However, patients with a PIV ≥ 488 had lower use of renin-angiotensin-system inhibitors (62% vs. 78%, *p* = 0.031) and a trend toward less frequent statin use (54% vs. 69%, *p* = 0.065). Diuretic use was significantly higher in the PIV ≥ 488 group (64% vs. 42%, *p* = 0.012).

Regarding the clinical outcomes, patients with PIV ≥ 488 experienced significantly higher one-year all-cause mortality (58% vs. 4%, *p* < 0.001) and in-hospital mortality (21% vs. 2%, *p* < 0.001). Rehospitalization rates were markedly higher (29% vs. 4%, *p* < 0.001), as were ischemic cerebrovascular events (12% vs. 4%, *p* < 0.001). Major bleeding complications were also more frequent among patients with higher PIV (10% vs. 2%, *p* = 0.034). The need for permanent pacemaker implantation and major vascular complications did not differ significantly between the groups.

**Figure 1:**
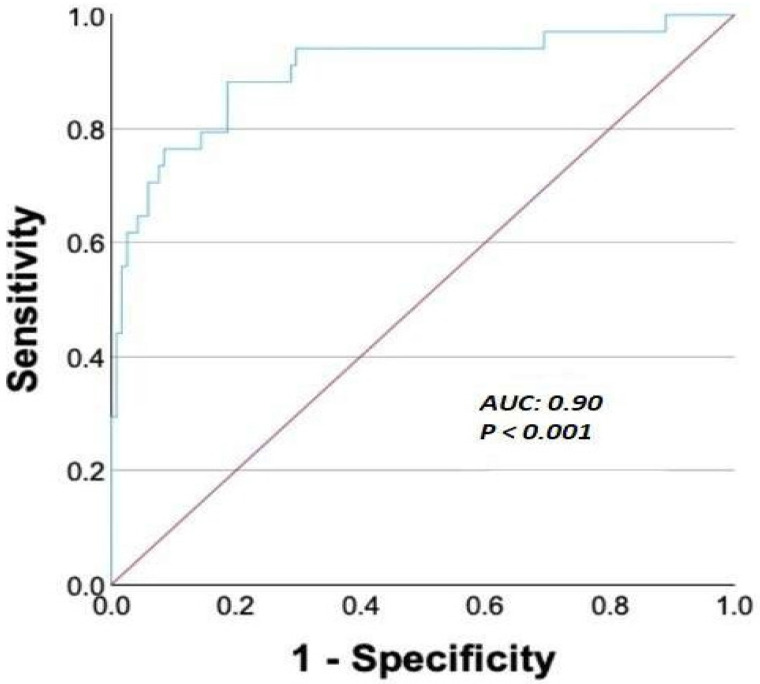
Receiver operating characteristic curve demonstrating the performance of the pan-immune inflammation value for predicting the all-cause mortality in patients undergoing TAVI. AUC = area under curve.

The multivariable logistic regression analysis identified age (OR: 1.108; 95% CI: 1.010–1.217; *p* = 0.031) and PIV (OR: 1.006; 95% CI: 1.003–1.008; *p* < 0.001) as independent predictors of all-cause mortality (Table 4). The predictive accuracy of the PIV was excellent, with an area under the curve (AUC) of 0.90 (*p* < 0.001) (Figure 1). A PIV cutoff of ≥488 demonstrated a sensitivity of 88% and specificity of 81% for predicting the all-cause mortality.

**Figure 2:**
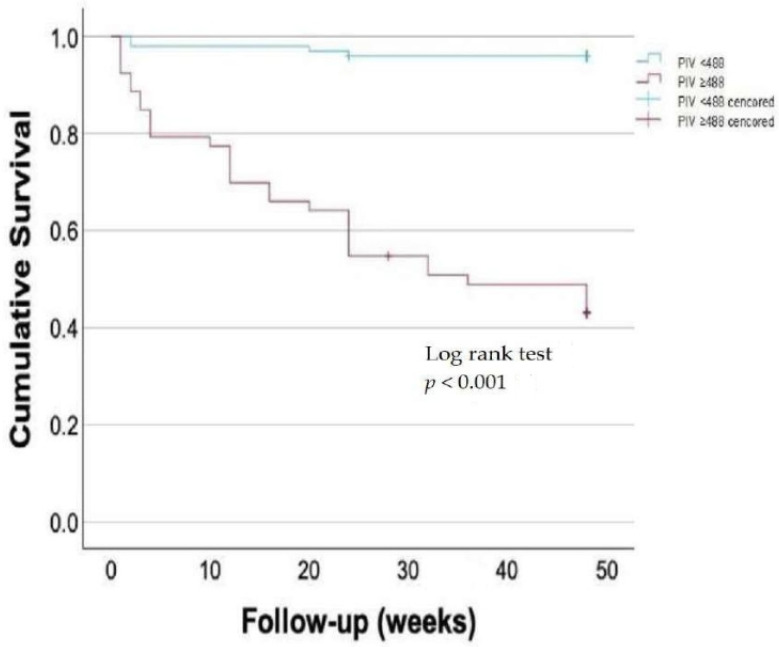
Kaplan–Meier survival plot showing an early and marked divergence in survival curves among patients stratified by the pan-immune inflammation value.

The Kaplan–Meier survival analysis demonstrated a significant difference in survival between patients stratified by the PIV (Figure 2). The patients in the high PIV group had markedly lower survival rates compared with those in the low PIV group (log rank test *p* < 0.001). The survival curves showed an early and persistent divergence.

## 4. Discussion

In our study, we demonstrated that the PIV is significantly associated with adverse clinical outcomes in patients with AS undergoing TAVI. To the best of our knowledge, this is the first study to assess the prognostic value of PIV in this population. Our findings indicate that elevated preprocedural PIV is independently linked to an increased risk of MACEs, in-hospital mortality, and one-year all-cause mortality.

Endothelial dysfunction and chronic inflammation, both of which are central to the pathogenesis of atherosclerosis, are also implicated in the progression of severe AS [13,14]. Currently, surgical valve replacement remains the standard treatment for AS patients with a low or moderate surgical risk [15]. In contrast, TAVI is predominantly performed in patients with a high surgical risk. The traditional risk scores used for TAVI, such as the Society of Thoracic Surgeons (STS) score and the European System for Cardiac Operative Risk Evaluation (EuroSCORE), fail to incorporate key factors like nutritional status, frailty, and inflammation, each of which is known to significantly impact mortality risk in AS patients undergoing TAVI [14,16]. Therefore, it is evident that additional prognostic parameters are necessary to enhance the risk assessment in this patient population.

Our results are consistent with previous studies reporting elevated CRP, neutrophil, and monocyte counts, as well as decreased lymphocyte counts, in patients who experienced MACEs [17,18]. Chronic inflammation has been widely implicated in adverse outcomes following TAVI. Condado et al. demonstrated that the NLR and platelet-to-lymphocyte ratio are effective markers for risk stratification in AS patients undergoing TAVI [19]. Similarly, Iglesias-Alvarez et al. found that an elevated baseline high-sensitivity CRP was an independent predictor of mortality post-TAVI [20].

Extending these prior findings, our study highlights the superior prognostic utility of PIV, a composite inflammatory index that incorporates neutrophil, monocyte, platelet, and lymphocyte counts, compared with individual inflammatory markers. Interestingly, CRP did not retain statistical significance in our multivariate logistic regression analysis, diverging from some earlier studies. However, the PIV remained a significant and independent predictor of mortality.

In a similar study conducted by Tosu et al., including 120 TAVI patients, the systemic immune-inflammation index (SII) was found to be a significant predictor of MACEs and short-term mortality in severe AS, further confirming the prognostic value of comprehensive inflammatory indices such as PIV and SII [21]. Notably, in this study, CRP lost statistical significance compared with the systemic immune-inflammation index; this finding is consistent with the results of our own study. This suggests that although CRP is a widely recognized marker of systemic inflammation, its predictive power for adverse outcomes in severe AS may be overshadowed by other inflammatory indices such as PIV, which provides a more comprehensive view of the inflammatory milieu. The loss of statistical significance for CRP in the multivariate logistic regression analysis may be attributed to its relatively nonspecific nature and limited ability to capture the complex inflammatory responses in severe AS. It can be better reflected by composite indices such as PIV and SII.

The value of inflammatory markers in cardiovascular risk stratification has been well documented. In particular, NLR and PIV have shown strong associations with adverse outcomes in conditions such as acute coronary syndrome and heart failure [22,23]. These findings reinforce the potential of PIV as a practical and effective tool for evaluating risk in patients undergoing TAVI.

In addition to PIV, age also emerged as an independent predictor of mortality. These findings underscore the pivotal role of systemic inflammation in post-TAVI prognosis and suggest that PIV may serve as a valuable biomarker for preprocedural risk stratification.

Collectively, our findings support the integration of composite inflammatory markers such as PIV, NLR, and SII into routine clinical assessments. Their use may enhance clinicians’ ability to stratify risk, anticipate complications, and personalize treatment strategies for TAVI candidates.

### Limitations and Future Perspectives

Despite the strengths of our study, several limitations should be acknowledged. First, this was a single-center retrospective study, which may have introduced selection bias and limited the generalizability of our findings. Second, considering the relatively high number of variables included in the multivariable analysis, the sample size was relatively small. This limitation could potentially be addressed by extending the patient recruitment period beyond two years or by designing a multicenter study. A multicenter approach would not only increase the sample size but also reduce the risk of selection bias. Third, although we observed a strong association between elevated PIV and adverse outcomes, causality cannot be established due to the retrospective nature of the study. Elevated PIV levels may reflect underlying comorbidities rather than acting as an independent predictor of mortality. Additionally, we did not assess the changes in PIV levels following TAVI, which might have provided further insights into the dynamic inflammatory response after the procedure. Therefore, our findings should be interpreted with caution and considered as a promising basis for future investigations rather than definitive evidence. Prospectively designed, large-scale multicenter studies are warranted to validate the prognostic value of PIV and to better define its potential role within a broader multimodal risk assessment strategy that integrates imaging, functional evaluation, and molecular biomarkers for patients undergoing TAVI.

## 5. Conclusions

In conclusion, our study identifies preprocedural PIV as a strong and independent predictor of all-cause mortality in patients undergoing TAVI. An elevated PIV reflects heightened systemic inflammation and is associated with adverse short- and long-term outcomes. Due to its simplicity, cost effectiveness, and high predictive accuracy, the PIV holds promise as a practical tool for preprocedural risk assessment.

## Figures and Tables

**Table 1:** Demographics, comorbidities, echocardiographic characteristics, and cardiac surgery risk of the study population stratified by the pan-immune inflammation value.

	All Patients (n = 152)	PIV ≥ 488 (n = 52)	PIV < 488 (n = 100)	*p* Value
Age, y	77 ± 7	77 ± 8	77 ± 7	0.99
Female sex, %	38 (25.0)	16 (31)	22 (22.0)	0.24
BMI, kg/m^2^	28.9 ± 5.5	29.0 ± 6.1	28.8 ± 5.2	0.87
Hypertension	132 (86.8)	48 (92)	84 (84.0	0.15
Diabetes mellitus, %	70 (46.1)	32 (62)	38 (38.0)	0.006
Hyperlipidemia, %	114 (75.0)	22 (65)	92 (78)	0.12
Coronary artery disease, %	100 (65.8)	32 (62)	68 (68.0)	0.43
Peripheral artery disease, %	11 (7.2)	5 (10)	6 (6)	0.41
History of cerebrovascular disease	10 (6.6)	5 (10)	5 (5.0)	0.28
Chronic kidney disease, %	28 (18.4)	16 (31)	12 (12.0)	0.005
Chronic obstructive lung disease, %	31 (20.4)	16 (31)	15 (15.0)	0.022
Atrial fibrillation	41 (27.0)	15 (29)	26 (26.0)	0.71
LVEF, %	53 ± 10	53 ± 11	53 ± 10	0.84
Transaortic mean gradient, mmHg	48 ± 13	46 ± 11	49 ± 13	0.13
Aortic valve area, cm^2^	0.7 ± 0.2	0.7 ± 0.2	0.7 ± 0.2	0.91
Bicuspid aortic valve, %	5 (3.3)	0 (0)	5 (5.0)	0.10
Aortic valve calcium score on CT (Hounsfield units)	2575 ± 1317	2594 ± 1630	2629 ± 1935	0.92
Valve-in-valve procedure, %	1 (0)	0 (0)	1 (0)	0.47
STS score	12.9 [9.8–18.3]	16.3 [10.8–19.7]	11.7 [9.5–15.2]	0.003

Abbreviations: BMI: body mass index; CT: computed tomography; LVEF: left ventricular ejection fraction; PIV: pan-immune inflammation value; STS score: Society of Thoracic Surgeons risk score.

**Table 2:** Laboratory parameters of the study population stratified by the pan-immune inflammation value.

	All Patients (n = 152)	PIV ≥ 488 (n = 52)	PIV < 488 (n = 100)	*p* Value
Leucocytes/μL	6935 (5508–8560)	8730 (6773–10,115)	6330 (5228–7558)	<0.001
Hemoglobin, g/dL	11 (9.6–12.2)	10.1 (9.3–11.6)	11.3 (10.0–12.4)	<0.001
Platelets × 10^3^, /μL	225 (174–275)	267 (210–316)	214 (164–253)	<0.001
Lympocytes/μL	1715 (1273–2150)	1330 (1083–1935)	1810 (1353–2315)	<0.001
Neutrophils/μL	4085 (3295–5278)	6275 (4453–7680)	3585 (2868–4290)	<0.001
Monocytes/μL	630 (505–760)	770 (593–913)	600 (480–670)	<0.001
Pan-immune inflammation value	340 (210–587)	760 (578–1041)	246 (165–339)	<0.001
Glucose, mg/dL	146 ± 60	168 ± 73	135 ± 49	0.001
Urea, mg/dL	55 ± 37	63 ± 37	51 ± 37	0.050
Creatinine, mg/dL	1.2 ± 0.6	1.4 ± 0.9	1.1 ± 0.4	0.002
Sodium, mEq/L	137 ± 4	137 ± 4	137 ± 4	0.25
Potassium, mEq/L	4.5 ± 0.4	4.6 ± 0.5	4.5 ± 0.4	0.36
C-reactive protein, mg/dL	6 (2–17)	15 (7–30)	4 (2–9)	<0.001

Abbreviations: PIV = pan-immune inflammation value.

**Table 3:** Medications, procedural features, and major adverse events of the study population stratified by the pan-immune inflammation value.

	All Patients (n = 152)	PIV ≥ 488 (n = 52)	PIV < 488 (n = 100)	*p* Value
Betablockers, %	113 (74.3)	37 (71)	76 (76.0)	0.52
Acetylsalicylic acid, %	101 (66.4)	30 (58)	71 (71.0)	0.099
Oral anticoagulant, %	34 (22.4)	14 (27)	20 (20.0)	0.33
Renin-angiotensin-system inhibitors, %	110 (72.4)	32 (62)	78 (78.0)	0.031
Statins, %	97 (63.8)	28 (54)	69 (69.0)	0.065
Diuretics, %	75 (49.3)	33 (64)	42 (42.0)	0.012
Predilatation, %	68 (44.7)	23 (44.2)	45 (45.0)	0.92
Postdilatation, %	55 (36.2)	20 (39)	35 (35.0)	0.67
Type of implanted valve				0.80
Self-expanding valve, %	118 (77.6)	41 (79)	77 (77.0)	
Balloon-expandable valve, %	34 (22.4)	11 (21)	23 (23.0)	
One-year all-cause mortality, %	34 (22.4)	30 (58)	4 (4.0)	<0.001
In-hospital mortality, %	13 (8.6)	11 (21)	2 (2.0)	<0.001
Need for permanent pacemaker, %	24 (15.8)	6 (12)	18 (18.0)	0.30
Major vascular access site complication, %	6 (3.9)	4 (8)	2 (2.0)	0.087
Major bleeding complication, %	7 (4.6)	5 (10)	2 (2.0)	0.034
Rehospitalization, %	19 (12.5)	15 (29)	4 (4.0)	<0.001
Ischemic cerebrovascular accident, %	10 (6.6)	6 (12)	4 (4.0)	<0.001

Abbreviations: PIV = pan-immune inflammation value.

**Table 4:** Multivariable logistic regression test for identifying the possible predictors of all-cause mortality.

	Odds Ratio	*p* Value
Age	1.108 (1.010–1.217)	0.031
Diabetes mellitus	0.803 (0.227–2.843)	0.73
Chronic kidney disease	1.263 (0.299–5.338)	0.75
Peripheral artery disease	0.271 (0.041–1.767)	0.17
Chronic obstructive lung disease	0.375 (0.090–1.557)	0.18
C-reactive protein	1.025 (0.996–1.054)	0.092
Pan-immune inflammation value	1.006 (1.003–1.008)	<0.001

## Data Availability

The raw data supporting the conclusions of this article will be made available by the authors on request.
